# Structured peptides as vaccine antigens against infections

**DOI:** 10.3389/fimmu.2026.1908831

**Published:** 2026-08-07

**Authors:** Claudia Trappetti, Hugo van Ingen, Mattia Falconi, Alice Romeo, Giada Sancamillo, Marco Caprini, Marco Rinaldo Oggioni, Marco Sette

**Affiliations:** 1 Department of Pharmacy and Biotechnology FABIT, University of Bologna, Bologna, Italy; 2 NMR Spectroscopy, Bijvoet Center for Biomolecular Research, Utrecht University, Utrecht, Netherlands; 3 Department of Biology, University of Rome “Tor Vergata”, Rome, Italy; 4 Bioinformatics Research Unit in Infectious Diseases, National Institute for Infectious Diseases “Lazzaro Spallanzani” – IRCCS, Rome, Italy; 5 European School of Molecular Medicine (SEMM), Milan, Italy; 6 Italian Institute for Genomic Medicine, c/o IRCSS Candiolo, Torino, Italy; 7 IRCCS Istituto delle Scienze Neurologiche di Bologna, Bologna, Italy; 8 IRCCS Azienda Ospedaliero-Universitaria di Bologna, Bologna, Italy; 9 INFN, University of Rome “Tor Vergata”, Rome, Italy; 10 CSPBAT Laboratory, Sorbonne Paris Cité, University of Paris 13, UMR 7244, CNRS, Bobigny, France; 11 Department of Chemical Sciences and Technology, University of Rome “Tor Vergata”, Rome, Italy

**Keywords:** disulfide loop, epitope grafting, epitope mimicry, *Pseudomonas aeruginosa*, type IV pili, vaccine

## Abstract

The onset of a new infection by a pathogen requires a rapid and prompt response from the body. Here, we present a simple and effective strategy to stimulate antibody production by grafting immunogenic peptides onto a beta-strand peptide scaffold. Analysis of the method using a known immunogenic region, the disulfide loop of the PilA protein of the *Pseudomonas aeruginosa* pilus, shows that the molecule folds properly in solution and, upon vaccination of mice, provides significant protection against intravenous challenge with the *P. aeruginosa* PAK strain.

## Introduction

Viral and bacterial infections are major problems in modern society (1) and ongoing challenges for the scientific community, which is why new tools are constantly sought to address them. Among bacterial pathogens, *Pseudomonas aeruginosa* is a particularly relevant model due to its clinical impact and treatment resistance. One of the methods studied in recent years involves grafting epitopes onto biological molecules to induce an immune response and protect against infections. Epitope grafting faces several challenges: the epitope must maintain its tertiary conformation to elicit the proper antibody response; the scaffold must also be tolerated by the body; and, finally, the system must be simple to prepare and, ideally, cost-effective, as it is planned for large-scale use.

Several groups have explored the use of proteins as scaffolds, which have the advantage of being stable molecules. However, such a system requires recombinant overproduction of the resulting molecule, followed by one or more purification steps, making this procedure lengthy and, in some cases, problematic. Furthermore, insertion of the epitope results in a chimeric protein that may fold incorrectly (2).

The use of peptides as scaffolds can overcome this problem, but peptides are inherently random coils. More recently, scaffold peptides containing disulfide bonds have been proposed to provide rigid ends that can accommodate an exogenous region (3).

In this work, we demonstrate that a small synthetic peptide, known as tryptophan-zipper (trpzip), is ideal for this purpose.

trp-zip is a class of small peptides, some of which can form a stable and very resistant β-hairpin secondary structure (4). The N- and C-terminal regions can be used to link external molecules, combining the structural rigidity of this short scaffold sequence with an immunogenic sequence, producing attractive templates for epitope presentation. Furthermore, since it is a short peptide, its synthesis is a low-cost process, an undoubted advantage over current methods for constructing monoclonal antibodies. trpzip2, the one used in this work, spontaneously folds into a β-hairpin, and the presence of four tryptophans interacting with each other results in an impressive increase in stability, with a resulting melting temperature of 72 °C, i.e., like a globular protein.

We then linked an immunogenic region at the C-terminal end of this scaffold. The immunogenic region chosen is the disulfide loop of the PilA protein of the PAK strain of *Pseudomonas aeruginosa*.

*P. aeruginosa* is ubiquitous in the environment but also an opportunistic human pathogen responsible for a wide range of infections. In people with cystic fibrosis, *P. aeruginosa* is the leading cause of chronic and relapsing pulmonary infections (5). Adherence of *P. aeruginosa* to the epithelial lining cells in the lung is mediated by several molecules, including both adhesins and type IV pili (6). Type IV pili are multi-subunit macromolecular structures assembled in filaments in which the most abundant protein is represented by the pilin PilA, in which the C-terminal region is a loop exposed to the solvent and closed at both ends by a disulfide bridge (PDB id: 5VXY) (7), called the disulfide loop. The loop spans 14–33 residues across different strains and has been shown to be immunogenic (8, 9).

Furthermore, synthetic peptides corresponding to the disulfide loop inhibit pili binding to the epithelial glycolipid (10).

## Materials and methods

### Computational studies

The peptide sequence was manually designed to obtain a specific fold and then submitted to a locally installed version of the AlphaFold2 program (11) in FASTA format to validate the requested fold. Since AlphaFold3 was publicly released only after the beginning of this study, we cross-checked our model against those generated with AlphaFold3, confirming high structural similarity in both the prediction of the trpzip2 motif and the loop arrangement. The obtained model was then simulated using the Gaussian accelerated molecular dynamics (GaMD) method (12) implemented in the AMBER22 program (13). Topology and coordinate files for the simulations were generated using the tleap module of AmberTools22 (14) and the AMBER ff19SB force field (15). Each peptide was placed in a box of TIP3P water molecules (16) and neutralised with NaCl ions, with a minimum distance of 14.0 Å from the box edges. To remove unfavourable interactions, two minimisation cycles were performed: each cycle consisted of 1000 steps of steepest descent minimisation, followed by 1000 steps of conjugate gradients. An initial restraint of 5 kcal·mol^-1^·Å^2^ was imposed on peptide atoms and then removed in the second cycle. Temperature was increased from 0 to 300 K in an NVT ensemble using Langevin dynamics (17) over 2.0 ns, with a timestep of 2 fs. A starting restraint of 0.5 kcal·mol^-1^·Å^2^ was applied to peptide atoms, then gradually decreased to relax the system. The system was then simulated in an isobaric-isothermal (NPT) ensemble for 50 ns, fixing the pressure to 1.0 atm using the Berendsen barostat (18) and maintaining the temperature to 300 K. GaMD equilibration was then performed for 100 ns (including 2 ns of preparatory classical MD steps), followed by 500 ns of GaMD production simulations. The simulations were performed in triplicate. The SHAKE algorithm (19) was used to constrain covalent bonds involving hydrogen atoms. The system’s coordinates were written every 5000 steps. Long-range interactions were calculated using the PME method (20), and an 8.0 Å cutoff was used for short-range interactions.

Structural analyses were performed on the trajectories to evaluate the conformational space explored by the peptides and the surface exposure of the PAK antigenic disulfide loop. RMSD, principal component analysis (PCA), and secondary structures were calculated using GROMACS 2023 (21). Solvent accessible surface area (SASA) for the disulfide loops was computed using the cpptraj tool of AmberTools22.

### Spectroscopic studies

The peptide was obtained from Caslo with a purity of 98% and a preformed disulfide bridge. Purification was checked by NMR spectroscopy.

Peptide concentration was determined from absorbance at 280 nm using an extinction coefficient of 22125 M^-1^ cm^-1^ (22).

CD spectra were collected on a Jasco J-1500 spectropolarimeter (JASCO Corporation, Tokyo, Japan), equipped with a PTC 510 Peltier system for thermal denaturation studies.

Samples were dissolved in 25 mM potassium phosphate buffer at pH 6.5.

For each spectrum, 4 scans were collected with an optical path length of 0.1 cm, a scan interval of 190–260 nm, a sampling interval of 0.2 nm, a scanning speed of 50 nm/min, and a bandwidth of 2 nm. Spectra were collected at 25 °C and 35 °C and reported as molar ellipticity. Melting experiments were conducted over a temperature range of 10 °C to 90 °C.

Data analysis was performed in MATLAB, and a two-state denaturation model was assumed (23).

NMR spectra were obtained on a Bruker Avance-NEO 900, operating at 900 MHz and equipped with a cryoprobe.

Samples were dissolved in 25 mM potassium phosphate buffer at pH 6.5 and measured at different temperatures. Optimal spectra were obtained at 35 °C, and this temperature was used for further analysis. Clean-TOCSY (60 ms), NOESY (150 ms), and natural abundance ^13^C-HSQC were collected to assign proton and carbon resonances and to obtain the relevant distance constraints from NOESY and backbone and χ_1_ angles from carbon chemical shifts by using the TALOS software (24) supplemented with a script kindly provided by Dr. Yang (Laboratory of Chemical Physics, National Institutes of Health, Bethesda). Spectra were processed with NMRPipe (25, 26) and data analysis was performed with NMRFAM-SPARKY (27). Protons and carbon assignments were deposited in the BMRB databank (accession code 35075). The restraints were used for the subsequent CYANA structure calculations (28) and the final structures were deposited in the PDB databank (accession code 32MP). The figure was drawn with CHIMERA software (29).

### *In vivo* studies

#### Peptide preparation

The trpzip2PAK peptide was provided in lyophilised form (2.9 mg total). The peptide was reconstituted in 1 mL of sterile phosphate-buffered saline (PBS; pH 7.4) under sterile conditions until complete solubilization. The stock solution was aliquoted and stored at −20 °C until use to avoid repeated freeze–thaw cycles. Immunisation doses were prepared to deliver 10 µg of peptide per mouse per administration.

Aluminium phosphate (0.5% w/v; 5 mg/mL) was used as an adjuvant for the peptide delivery, diluted 1:10 in sterile PBS to obtain a final working concentration of 0.5 mg/mL.

Immediately prior to immunisation, trpzip2PAK was mixed with aluminium phosphate at a 10:1 (adjuvant: peptide, w/w) ratio. The formulation was gently vortexed and incubated at room temperature for 15 minutes to allow peptide adsorption onto the adjuvant.

Control formulations consisted of adjuvant alone, prepared under identical conditions.

#### Animals and experimental design

Female C57BL/6 mice (6–8 weeks old) were used in all experiments. Animals were housed under specific pathogen-free (SPF) conditions with ad libitum access to food and water.

Mice were randomly assigned to experimental groups (n = 30 per group). For each formulation, the following groups were included:

1. Vaccinated group: trpzip2PAK formulated with aluminium phosphate
2. Control group: aluminium phosphate alone

All animal procedures were conducted at the university of Bologna in accordance with licence n° n. 174/2025-PR and were approved by the Italian Ministry of Health.

#### Immunization protocol

Mice were immunised subcutaneously according to the following schedule:

- Day 0: primary immunisation
- Day 21: booster immunization
- Day 35: second booster immunisation

Each mouse received 10 µg of trpzip2PAK per immunization in a total injection volume of 100 µL.

The total amount of peptide administered per vaccinated group was 300 µg (10 µg × 10 mice × 3 immunisations). Animals were monitored daily after each immunisation for general appearance, body weight, grooming behaviour, mobility and signs of local reactogenicity at the injection site. No adverse effects were observed.

Blood samples were collected from the submandibular vein prior to immunisation (baseline) and two weeks after the final booster for serological analysis.

#### Intravenous infection model

Two weeks after the final immunisation, mice were intravenously challenged with *P. aeruginosa* PAK at a dose of 5x10^7^ CFU/ml. At 24h post-infection, blood was collected, and bacterial load was quantified.

Blood was serially diluted in sterile PBS and plated on appropriate agar plates. Colony-forming units (CFU) were quantified after incubation, and bacteraemia was defined as the presence of detectable CFU in blood samples.

#### ELISA for anti-trpzip2PAK IgG

Total anti-trpzip2PAK IgG levels were measured by enzyme-linked immunosorbent assay (ELISA).

Briefly, 96-well plates were coated overnight at 4 °C with trpzip2PAK (carbonate-bicarbonate buffer). Plates were washed and blocked with 1% BSA in PBS for 1 hour at room temperature.

Serial dilutions of mouse sera were added and incubated for 2 hours at room temperature. After washing, HRP-conjugated anti-mouse IgG secondary antibody was added and incubated for 1 hour. Plates were developed using TMB substrate, and the reaction was stopped with 1 M H_2_SO_4_. Optical density (OD) was measured at 450 nm using a microplate reader.

#### Statistical analysis

Data are presented as mean ± standard error of the mean (SEM) unless otherwise indicated. Differences between groups were analysed using the Mann–Whitney U test. A p-value < 0.05 was considered statistically significant. Statistical analyses were performed using GraphPad Prism.

## Results

### Computational studies

The trpzip2 motif has been used to design one molecule in which the C-terminal end of the trpzip2 is directly linked to the N-terminal end of the disulfide loop from the *P. aeruginosa* PAK strain, resulting in the trpzip2PAK peptide.

The final peptide sequence is: swtwengkwtwkCTSDQDEQFIPKGC (26 amino acids). Here, lowercase letters refer to the trpzip2 scaffold (4), while uppercase letters refer to the disulfide loop of PilA (residues 135-148) from the *P. aeruginosa* PAK strain.

**Figure 1:**
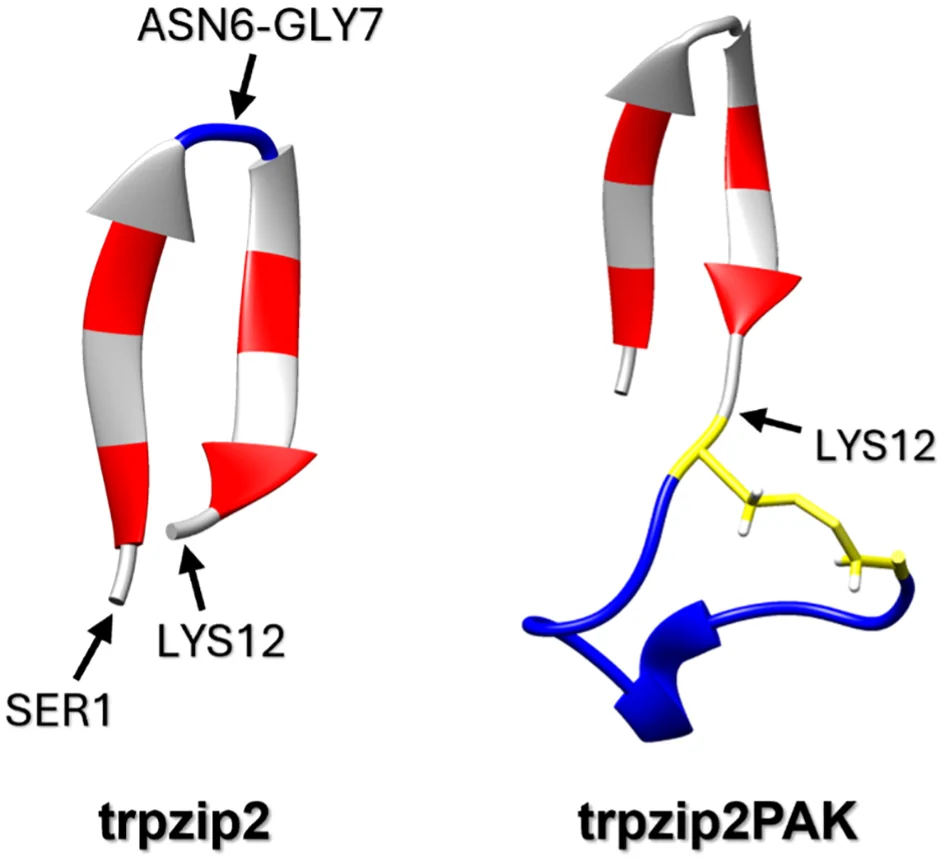
The structure of the trpzip2 scaffold (left) and the engineered trpzip2PAK peptide (right). The residues of trpzip2 at the PAK loop insertion sites are noted. The loop regions are highlighted in blue, while tryptophan residues are depicted in red. Cysteines participating in disulfide bridges are shown as yellow sticks.

The final peptide contains 26 amino acids, with the PAK loop closed at the edge by the two cysteines present in the wild-type PAK pilus protein. The peptide was modelled using the AlphaFold2 software (11), which correctly placed the antigenic disulfide loop and exposed it to solvent (**Figure 1**).

To assess the peptide’s stability and conformational flexibility, molecular dynamics (MD) simulations were performed. Three 500 ns MD simulation replicas were performed using the Gaussian-accelerated Molecular Dynamics (GaMD) method (12) and the AMBER22 program (13) to explore a larger conformational space.

**Figure 2:**
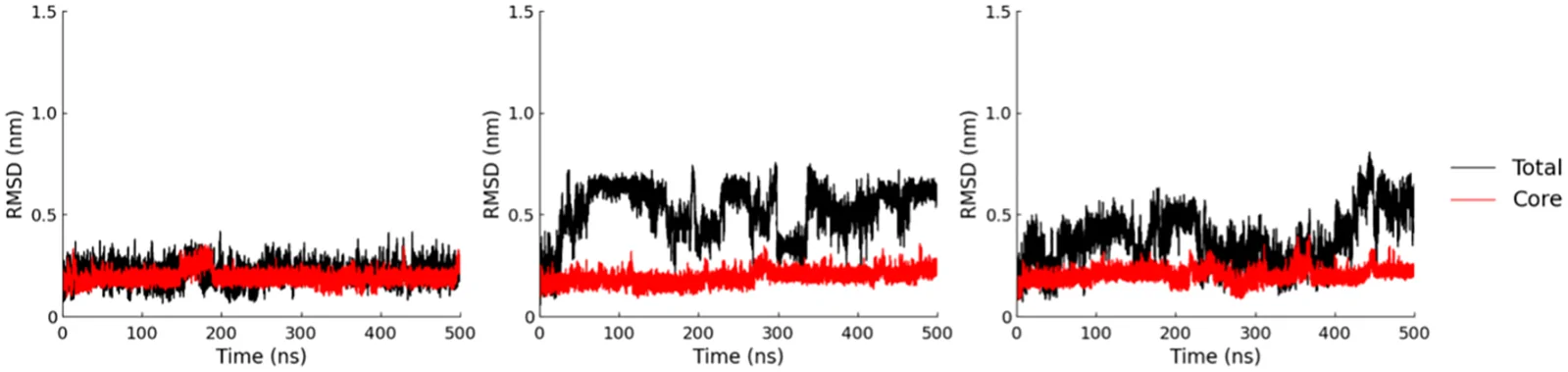
RMSD calculated on total Cɑ atoms (‘total’, i.e., the complete peptide structure) and on the Cɑ of the scaffold core (‘core’, i.e., the trpzip2 motif excluding the loop) for each peptide replica.

The RMSD analysis to evaluate the peptide’s overall stability during the simulations (**Figure 2**) was calculated for the C_α_ atoms of the complete structure (‘total’) and the scaffold core (‘core’, i.e., the trpzip2 motif excluding the loop). This analysis indicates that the peptide structure is stable during the simulations, with total RMSD peaks below 1 nm. In one replica, the total RMSD closely matches that of the scaffold core, suggesting that the loop is rigid; in the other two replicas, the loop regions have a greater impact on the RMSD but do not cause substantial fluctuations.

Secondary structure was calculated during all trajectories (**Supplementary Figure 1**, **Supplementary Material**). Importantly, the scaffold β-strands maintain their secondary structure throughout all simulations, confirming their stability. On the other hand, as expected, the loop residues remain largely flexible, exploring different bends, turns, and loop configurations, with a solvent exposure of 85-95% across all three replicas. In two of three replicas, transient α-helical formation within the loops is also observed.

To determine the extent of the conformational space explored by the peptide, principal component analysis (PCA) of motion was performed for all trajectories (30). Using the GROMACS 2023 (21) software, a covariance matrix was generated for all residue pairs of each peptide. Diagonalisation of the covariance matrix yielded the eigenvectors and corresponding eigenvalues that describe the system’s principal motions. The first eigenvector accounts for 29-45% of the total motion, while the second accounts for 15-23%. This means the first two components explain about 45-67% of the total motion and were therefore selected for further analysis.

**Figure 3:**
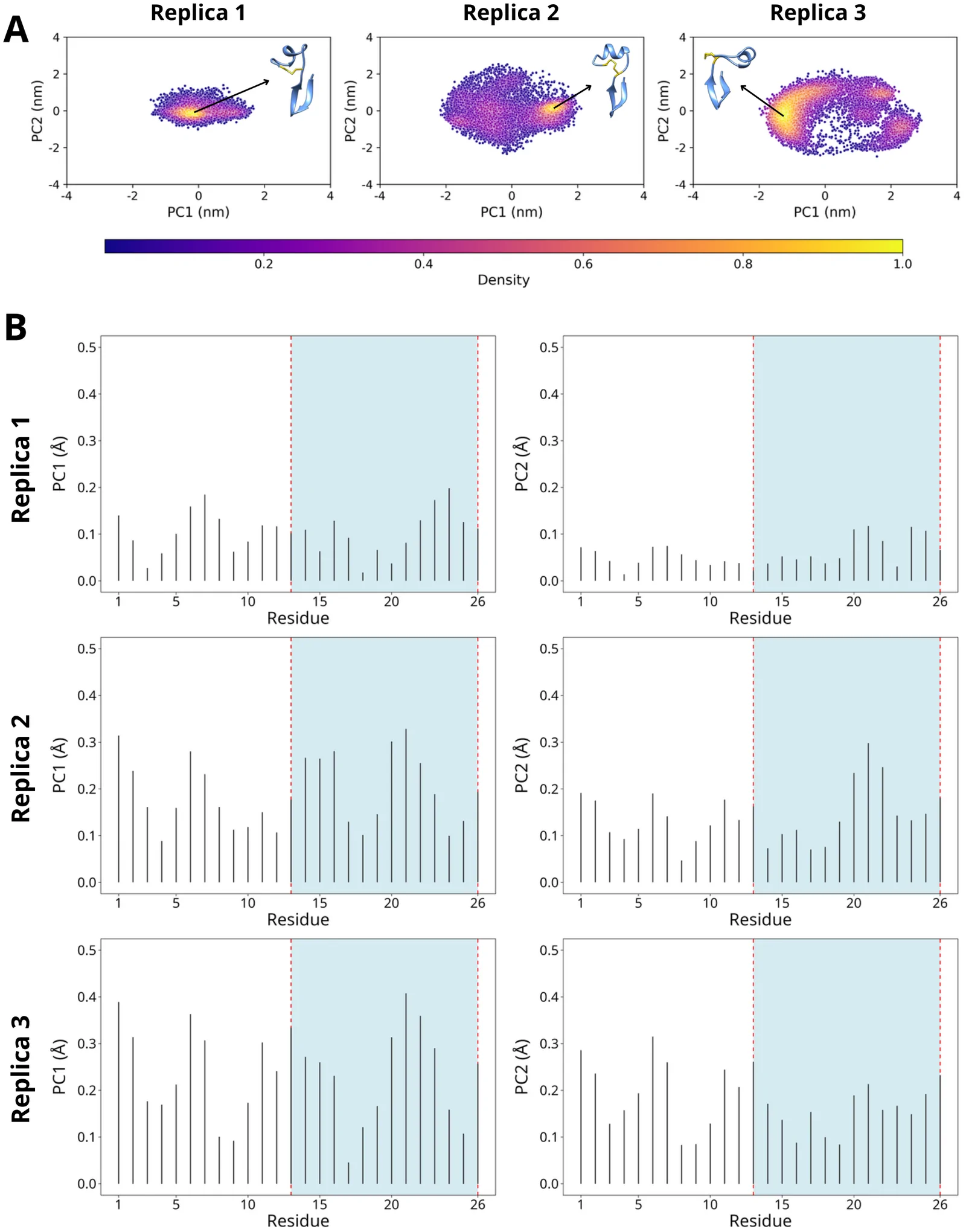
**(A)** Projections of the first two eigenvectors resulting from PCA of trpzip2PAK trajectories, corresponding to the conformational space explored by the peptide in each simulation replica. The legend indicates the KDE density estimated for the projected data. The most stable peptide conformation for each replica is also represented. **(B)** Per-residue loadings (i.e., contributions) in Å to the first two principal components. The red dashed lines separate the peptide’s loop (in cyan background) from the core.

In **Figure 3A**, the projections of the first two principal components onto the conformational space are shown. Kernel density estimation (KDE) was applied to the projected data to identify regions of high point density, corresponding to energetically stable or frequently visited states during the simulations. In two of the three replicas, broader conformational sampling and more diffuse density distributions were observed. Instead, one replica shows more restricted sampling with distinct high-density clusters, indicative of stable, well-defined conformations throughout the simulations. The space probed by the three replicas, although broad, appears to be nearly superimposable. As shown in the RMSD results (**Figure 2**), the higher conformational sampling in the second and third replicas is mostly due to loop flexibility. This is also confirmed by residue-wise loadings (i.e., contributions) to the first and second principal components, which indicate the regions contributing most to the structure’s dominant modes of motion. High loading values identify residues whose movements are strongly correlated with the corresponding eigenvector, i.e., residues having the highest fluctuations during the simulations (**Figure 3B**). The results confirm that the loop is flexible in this peptide. However, some flexibility is also observed in the β-strands for the second and third replicas.

### Spectroscopic analysis

*In vitro* folding was assessed by performing circular dichroism (CD) and NMR spectroscopy.

**Figure 4:**
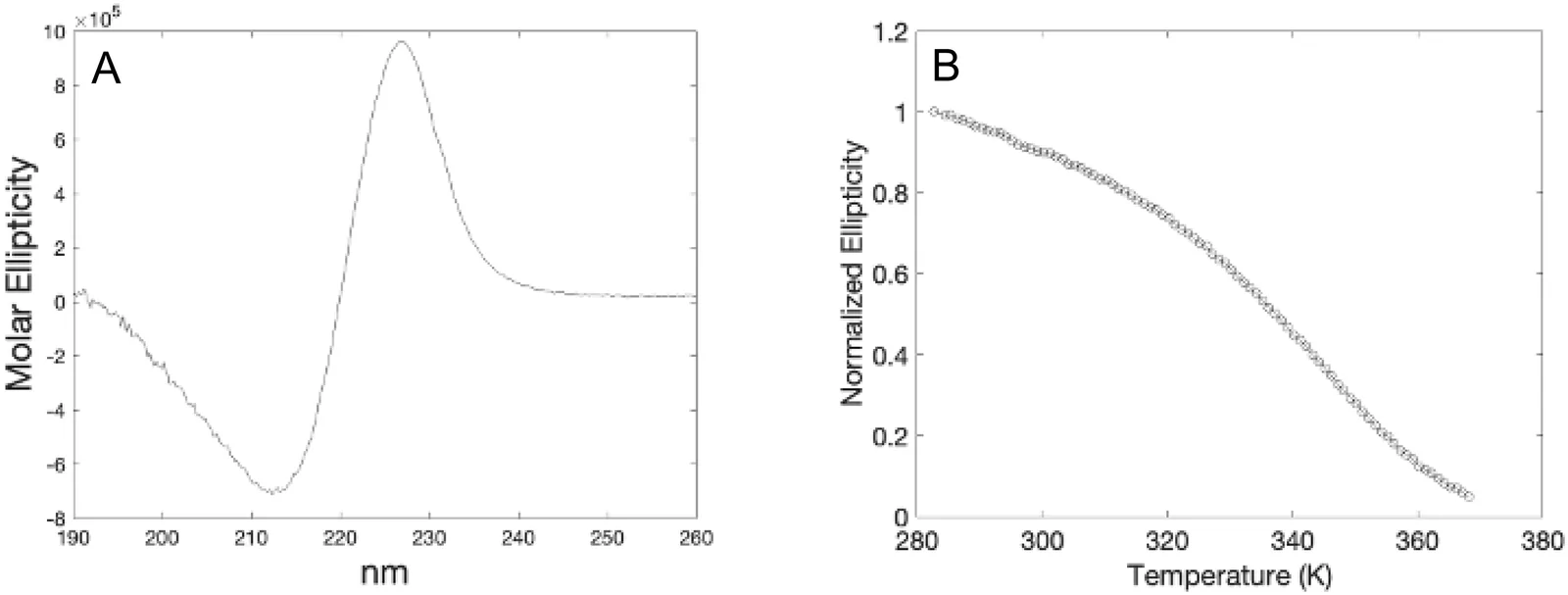
CD analysis of trpzip2PAK peptide. **(A)** Far-UV spectrum of trpzip2PAK showing the typical bands of trpzip2 at 227 and 212 nm; **(B)** Thermal denaturation of trpzip2PAK obtained by following the decrease of the 227 nm band with increasing temperature.

The CD spectrum of trpzip2PAK is shown in **Figure 4A**. The two bands at 227 and 212 nm result from stacking interactions among the four tryptophans, as observed in trpzip2 (4), suggesting that the scaffold folds properly.

Since disordered conformations typically produce a signal around 200 nm, the intensity of the 212 nm signal may reflect the presence of a loop adopting a disordered conformation.

Melting denaturation was measured by following the decrease in the 227 nm band as the temperature increased (**Figure 4B**). The results indicate a melting point of 61 °C, like that of trpzip2 (72 °C).

This ensures the peptide can be used over a wide range of temperatures.

Further *in vitro* characterisation was performed using NMR spectroscopy.

The spectrum shows sharp, well-resolved, dispersed signals, suggesting a folded structure. Homonuclear and heteronuclear 2D-NMR experiments allowed us to assign the proton and carbon resonances. Comparison between the chemical shifts of the scaffold protons of trpzip2PAK and the reported ones of trpzip2 shows that resonances having peculiar positions in trpzip2 (Trp2, Thr3, Trp9, Thr10) maintain the same positions in trpzip2PAK, suggesting an identical folding.

Distance and chemical-shift restraints were used for structure calculations with CYANA (28).

The final 20 conformers obtained have an average target function of 0.55 ± 0.01 Å^2^.

An RMSD of 0.04 ± 0.02 Å for the backbone and 0.26 ± 0.05 Å for the side chains in the structured region (0.68 ± 0.24 Å and 1.03 ± 0.29 Å, including the disulfide loop) was obtained.

Structural statistics (31) are reported in **Supplementary Table 1**.

**Figure 5:**
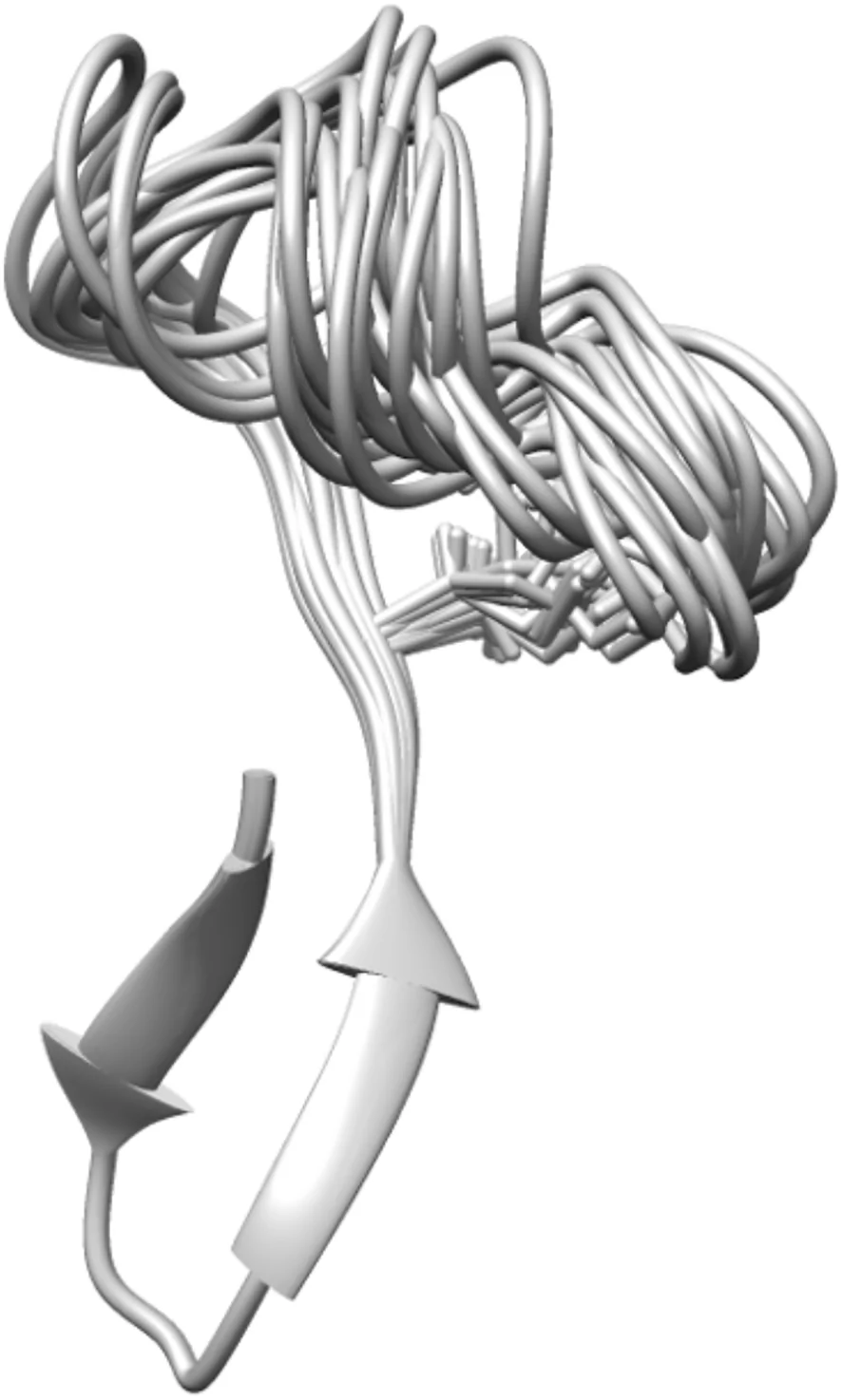
3D-NMR structure of trpzip2PAK peptide showing the scaffold region corresponding to trpzip2 at the bottom and the disulfide loop region at the top. The final 20 conformers from CYANA analysis are shown.

The 3D structure is reported in **Figure 5** and confirms the expected folding, with the scaffold properly folded and the loop maintaining the expected flexibility.

### *In vivo* experiments

**Figure 6:**
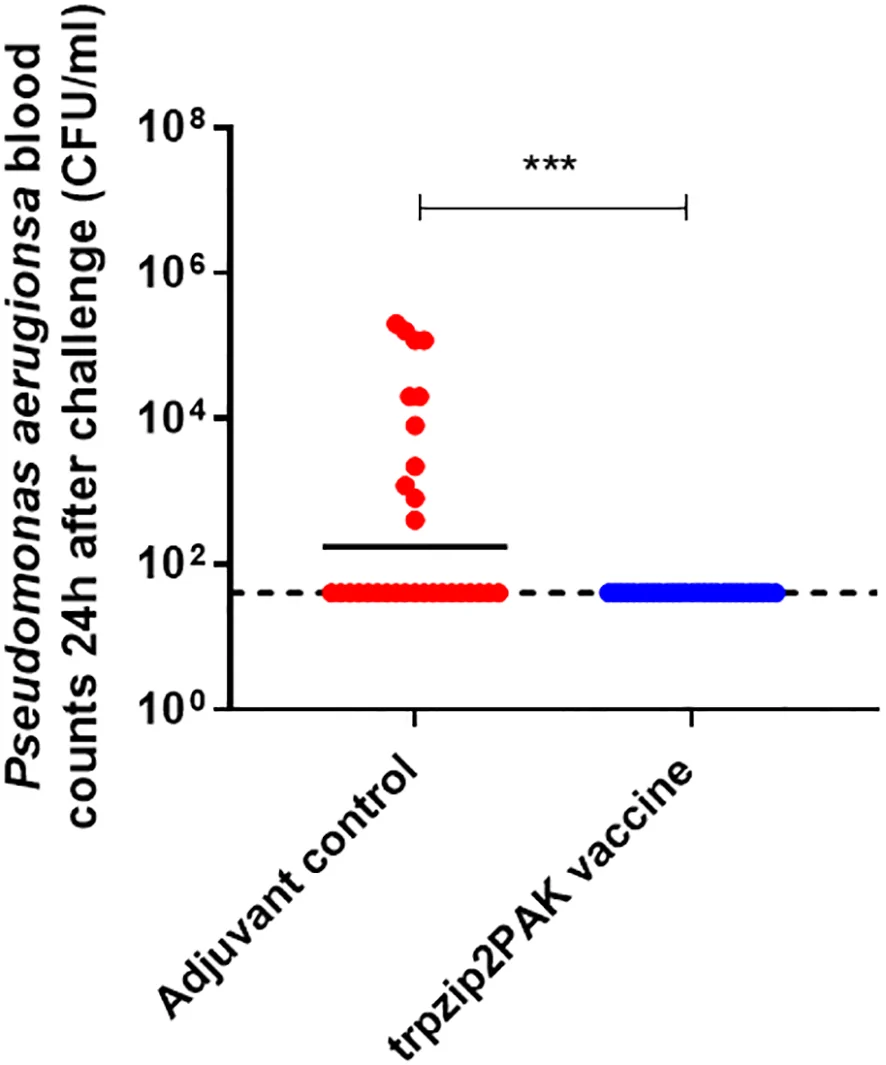
Efficacy of trpzip2PAK vaccine against *Pseudomonas aeruginosa* systemic infection. Bacterial burden in the blood of mice 24 hours after challenge with *P. aeruginosa*. Mice were immunised with either the adjuvant control (red dots) or the trpzip2PAK vaccine (blue dots). Data are expressed as Colony Forming Units per millilitre (CFU/ml) on a logarithmic scale. The horizontal bars represent the median values, and the dashed line indicates the assay’s limit of detection. Statistical significance was determined using the Mann-Whitney test, where *** indicates p < 0.001 (p = 0.0004).

To explore the potential of the structured peptides as vaccine candidates, we employed an acute murine *P. aeruginosa* bacteraemia model. Mice were immunised intramuscularly with 10 µg of trpzip2PAK per dose, administered at 0, 21, and 35 days, plus aluminium phosphate (alum) as an adjuvant, while a control group received alum only. At day 49, mice were intravenously challenged with *P. aeruginosa* PAK at a dose of 5 × 10^7^ CFU/ml. 24h post-infection, blood samples were collected, and bacterial load was determined. The results show that none of the mice immunised with trpzip2PAK had detectable bacteria in the blood (**Figure 6**).

In contrast, a proportion of mice in the alum-only control group were bacteraemic. Although several control animals did not show detectable bacteria in the blood, the difference between the groups remained significant. This is consistent with previous reports indicating that, in murine models of *P. aeruginosa* infection, intrinsic variability in the host response can be observed (32). Importantly, vaccination conferred clear protection, as none of the immunised animals showed detectable bacteria in the bloodstream.

**Figure 7:**
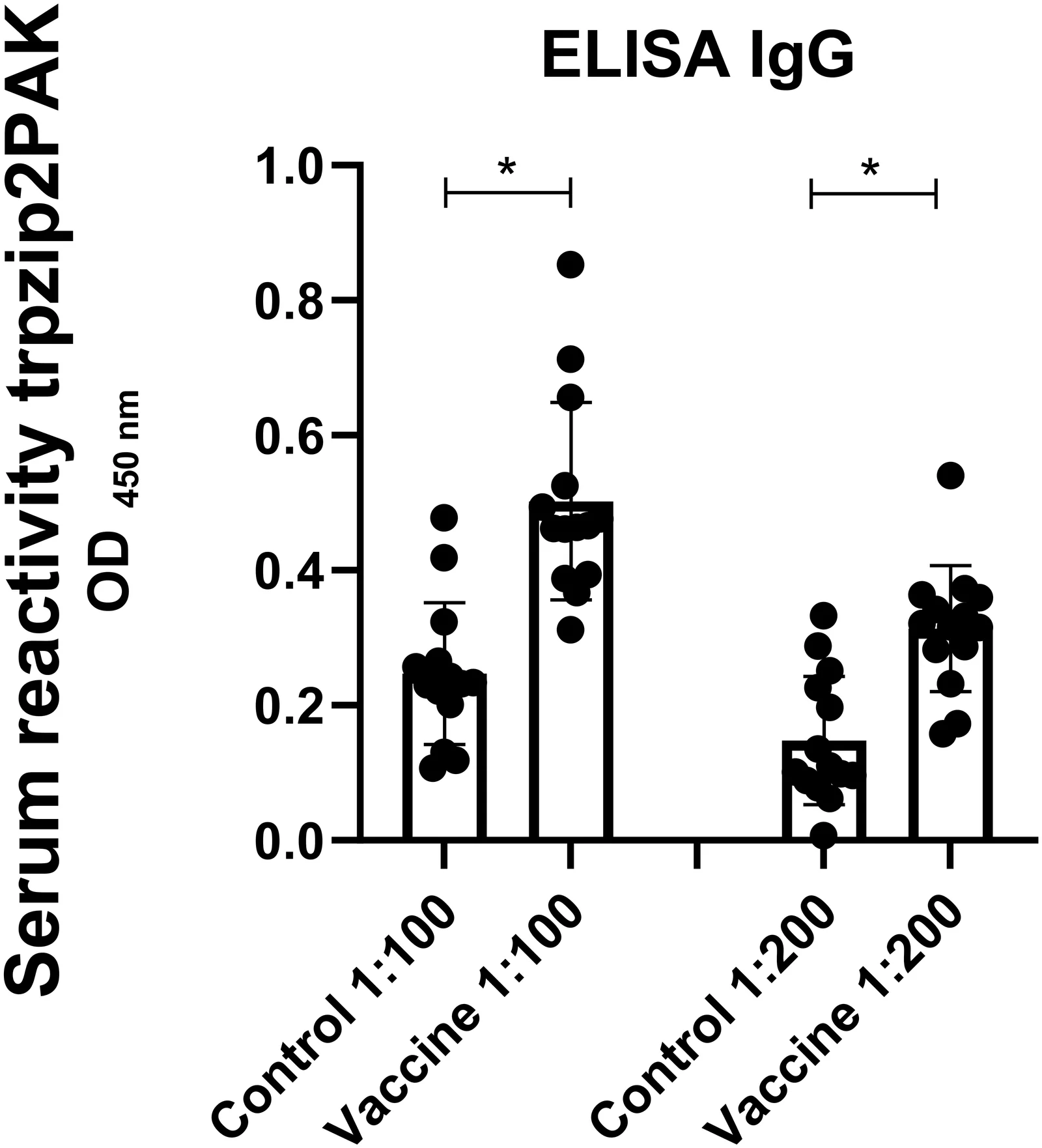
Serum IgG antibody responses measured by ELISA. Serum IgG levels in vaccinated and control mice were determined by enzyme-linked immunosorbent assay (ELISA) two weeks after the third immunisation. Antibody levels are expressed as absorbance values at 450 nm (OD_450_). Statistical comparisons between groups were performed using the Mann–Whitney U test. P values are indicated in the figure; p < 0.05 (p = 0.028) was considered statistically significant. Statistical significance was determined using the Mann-Whitney test, where * indicates p < 0.05 (p = 0.028).

Interestingly, despite relatively modest serum antibody titres in the vaccinated group (with detectable IgG at a 1:200 dilution, **Figure 7**), this response was sufficient to mediate rapid clearance of *P. aeruginosa* from the bloodstream. These findings suggest that even low anti-trpzip2PAK antibody titres can confer functional protection in our low-severity infection model. However, the current study does not allow us to determine the quality of the antibody response, including antibody affinity, avidity, or other effector functions. Overall, our results indicate that the designed peptide effectively mimics the antigenic properties of the native PilA loop and may therefore represent a promising vaccine antigen capable of preventing invasive Pseudomonas infection.

## Discussion

In this work, we have shown a novel, simple and low-cost strategy to elicit an immune response by using a structured peptide as a scaffold for an antigenic region. As a proof of concept, the grafting of the *Pseudomonas aeruginosa* PAK PilA disulphide loop onto a highly structured hairpin scaffold, such as that of the trpzip2 peptide, has been tested and shows that the resulting molecule is folded as expected and fully functional, conferring a distinct immunological advantage in antibody specificity. A further advantage is temperature stability over a wide range.

By anchoring the edges of the PAK loop to the rigid trpzip2 framework, the conformational entropy of the isolated epitope is drastically reduced, mimicking the native-like three-dimensional architecture. Consequently, B-cell receptors (BCRs) are exclusively exposed to disease-relevant conformation, driving the selection of highly specific antibodies and preventing the generation of cross-reactive clones typically associated with highly flexible, disordered peptides (2).

This approach proved successful *in vivo*, as mice immunised with trpzip2PAK exhibited a protective effect, showing improved control and resolution of *P. aeruginosa* bacteraemia.

The loop used in this work is 14 residues long, including the cysteines at the edges, but experiments in our laboratory show that loops of more than 30 residues can also be accommodated, making this strategy feasible in different situations, since an immunogenic loop can easily be linked at its edges by a disulfide bridge, provided the final conformation is not different from the *in vivo* one (33).

Furthermore, once the antibodies are produced, the peptide can be immobilised on a chromatographic column for affinity purification, and bound antibodies can then be eluted with solutions at different pH or ionic strength.

Concerning *Pseudomonas* infections, different clinical strains are present, and the disulfide loop region is not always conserved, but recent studies (34) indicate that the number of infectious clones is quite limited, thus suggesting that several peptides can be manufactured using the same technique, each with a disulfide loop of a representative strain, and the cocktail of peptides can elicit an immune response against a representative number of strains of the infection.

Furthermore, since it is known that PilA interacts with the asialo-GM1 moiety of epithelial cells through the disulfide loop (10), and *in vitro* data show competition between the disulfide loop and *Pseudomonas* against asialo-GM1, we cannot exclude that trpzip2PAK, and similar peptides with loops from a representative group of strains, also inhibit the binding of *P. aeruginosa* by competing for the same binding site, thus suggesting a dual role for such peptides. However, direct assessment of inhibition of PilA binding to asialo-GM1 was beyond the scope of the present proof-of-concept study. Therefore, while a possible dual role of the peptide as both an immunogen and an inhibitor of bacterial adhesion cannot be excluded, this remains speculative and requires dedicated experimental validation in future studies.

Overall, this approach provides a versatile platform for the rational design of peptide immunogens, with potential applications not only in anti-*Pseudomonas* strategies but also in targeting structurally defined epitopes in other pathogens or cancer cells.

## Data Availability

The original contributions presented in the study are included in the article/**Supplementary Material**. Further inquiries can be directed to the corresponding author.

## In memoriam

In memory of Professor Lorenzo Stella.

## References

[1] Jesudason T . Who publishes updated list of bacterial priority pathogens. Lancet Microbe. (2024) 5. doi: 10.1016/j.lanmic.2024.07.003 39079540

[2] Correia BE Bates JT Loomis RJ Baneyx G Carrico C Jardine JG . Proof of principle for epitope-focused vaccine design. Nature. (2014) 507:201–6. doi: 10.1038/nature12966 24499818 PMC4260937

[3] Yin H Craik DJ Wang CK . Anchor residues guide form and function in grafted peptides. Angew Chem Int Ed. (2019) 58:7652–6. doi: 10.1002/anie.201901572 30916847

[4] Cochran AG Skelton NJ Starovasnik MA . Tryptophan zippers: stable, monomeric beta -hairpins. Proc Natl Acad Sci USA. (2001) 98:5578–83. doi: 10.1073/pnas.091100898 11331745 PMC33255

[5] Rossi E La Rosa R Bartell JA Marvig RL Haagensen JAJ Sommer LM . Pseudomonas aeruginosa adaptation and evolution in patients with cystic fibrosis. Nat Rev Microbiol. (2021) 19:331–42. doi: 10.1038/s41579-020-00477-5 33214718

[6] Irvin RT . Adherence of pseudomonas aeruginosa. In: Pseudomonas. John Wiley & Sons, Ltd (2008). p. 45–83. doi: 10.1002/9783527622009.ch3

[7] Wang F Coureuil M Osinski T Orlova A Altindal T Gesbert G . Cryoelectron microscopy reconstructions of the Pseudomonas aeruginosa and Neisseria gonorrhoeae type IV pili at sub-nanometer resolution. Structure. (2017) 25:1423–1435.e4. doi: 10.1016/j.str.2017.07.016 28877506 PMC8189185

[8] Doig P Sastry PA Hodges RS Lee KK Paranchych W Irvin RT . Inhibition of pilus-mediated adhesion of Pseudomonas aeruginosa to human buccal epithelial cells by monoclonal antibodies directed against pili. Infect Immun. (1990) 58:124–30. doi: 10.1128/iai.58.1.124-130.1990 1967166 PMC258418

[9] Wong WY Irvin RT Paranchych W Hodges RS . Antigen-antibody interactions: elucidation of the epitope and strain-specificity of a monoclonal antibody directed against the pilin protein adherence binding domain of Pseudomonas aeruginosa strain K. Protein Sci. (1992) 1:1308–18. doi: 10.1002/pro.5560011010 1284654 PMC2142108

[10] Lee KK Sheth HB Wong WY Sherburne R Paranchych W Hodges RS . The binding of Pseudomonas aeruginosa pili to glycosphingolipids is a tip-associated event involving the C-terminal region of the structural pilin subunit. Mol Microbiol. (1994) 11:705–13. doi: 10.1111/j.1365-2958.1994.tb00348.x 7910938

[11] Jumper J Evans R Pritzel A Green T Figurnov M Ronneberger O . Highly accurate protein structure prediction with AlphaFold. Nature. (2021) 596:583–9. doi: 10.1038/s41586-021-03819-2 34265844 PMC8371605

[12] Wang J Arantes PR Bhattarai A Hsu RV Pawnikar S Huang YM . Gaussian accelerated molecular dynamics: principles and applications. WIREs Comput Mol Sci. (2021) 11:e1521. doi: 10.1002/wcms.1521 34899998 PMC8658739

[13] Case DA Cheatham TE Darden T Gohlke H Luo R Merz KM . The Amber biomolecular simulation programs. J Comput Chem. (2005) 26:1668–88. doi: 10.1002/jcc.20290 16200636 PMC1989667

[14] Salomon-Ferrer R Case DA Walker RC . An overview of the Amber biomolecular simulation package. WIREs Comput Mol Sci. (2013) 3:198–210. doi: 10.1002/wcms.1121 41531421

[15] Tian C Kasavajhala K Belfon KAA Raguette L Huang H Migues AN . Ff19SB: amino-acid-specific protein backbone parameters trained against quantum mechanics energy surfaces in solution. J Chem Theory Comput. (2020) 16:528–52. doi: 10.1021/acs.jctc.9b00591 31714766 PMC13071887

[16] Jorgensen WL Chandrasekhar J Madura JD Impey RW Klein ML . Comparison of simple potential functions for simulating liquid water. J Chem Phys. (1983) 79:926–35. doi: 10.1063/1.445869

[17] Goga N Rzepiela AJ de Vries AH Marrink SJ Berendsen HJC . Efficient algorithms for Langevin and DPD dynamics. J Chem Theory Comput. (2012) 8:3637–49. doi: 10.1021/ct3000876 26593009

[18] Berendsen HJC Postma JPM van Gunsteren WF DiNola A Haak JR . Molecular dynamics with coupling to an external bath. J Chem Phys. (1984) 81:3684–90. doi: 10.1063/1.448118

[19] Ryckaert J-P Ciccotti G Berendsen HJC . Numerical integration of the cartesian equations of motion of a system with constraints: molecular dynamics of n-alkanes. J Comput Phys. (1977) 23:327–41. doi: 10.1016/0021-9991(77)90098-5

[20] Darden T York D Pedersen L . Particle mesh Ewald: an N·log(N) method for Ewald sums in large systems. J Chem Phys. (1993) 98:10089–92. doi: 10.1063/1.464397 41411595

[21] Abraham MJ Murtola T Schulz R Páll S Smith JC Hess B . GROMACS: high performance molecular simulations through multi-level parallelism from laptops to supercomputers. SoftwareX. (2015) 1–2:19–25. doi: 10.1016/j.softx.2015.06.001 38826717

[22] Gill SC von Hippel PH . Calculation of protein extinction coefficients from amino acid sequence data. Anal Biochem. (1989) 182:319–26. doi: 10.1016/0003-2697(89)90602-7 2610349

[23] Minor DL Kim PS . Measurement of the beta-sheet-forming propensities of amino acids. Nature. (1994) 367:660–3. doi: 10.1038/367660a0 8107853

[24] Shen Y Delaglio F Cornilescu G Bax A . TALOS+: a hybrid method for predicting protein backbone torsion angles from NMR chemical shifts. J Biomol NMR. (2009) 44:213–23. doi: 10.1007/s10858-009-9333-z 19548092 PMC2726990

[25] Delaglio F Grzesiek S Vuister GW Zhu G Pfeifer J Bax A . NMRPipe: a multidimensional spectral processing system based on UNIX pipes. J Biomol NMR. (1995) 6:277–93. doi: 10.1007/BF00197809 8520220

[26] Maciejewski MW Schuyler AD Gryk MR Moraru II Romero PR Ulrich EL . NMRbox: a resource for biomolecular NMR computation. Biophys J. (2017) 112:1529–34. doi: 10.1016/j.bpj.2017.03.011 28445744 PMC5406371

[27] Lee W Tonelli M Markley JL . NMRFAM-SPARKY: enhanced software for biomolecular NMR spectroscopy. Bioinformatics. (2015) 31:1325–7. doi: 10.1093/bioinformatics/btu830 25505092 PMC4393527

[28] Güntert P Buchner L . Combined automated NOE assignment and structure calculation with CYANA. J Biomol NMR. (2015) 62:453–71. doi: 10.1007/s10858-015-9924-9 25801209

[29] Pettersen EF Goddard TD Huang CC Couch GS Greenblatt DM Meng EC . UCSF Chimera--a visualization system for exploratory research and analysis. J Comput Chem. (2004) 25:1605–12. doi: 10.1002/jcc.20084 15264254

[30] Amadei A Linssen ABM Berendsen HJC . Essential dynamics of proteins. Proteins: Structure Function Bioinf. (1993) 17:412–25. doi: 10.1002/prot.340170408 8108382

[31] Bhattacharya A Tejero R Montelione GT . Evaluating protein structures determined by structural genomics consortia. Proteins. (2007) 66:778–95. doi: 10.1002/prot.21165 17186527

[32] Bragonzi A . Murine models of acute and chronic lung infection with cystic fibrosis pathogens. Int J Med Microbiol. (2010) 300(8):584–93. doi: 10.1016/j.ijmm.2010.08.012 20951086

[33] Di Vona ML Rossolini GM Sette M . A strategy based on loop analysis to develop peptide epitopes: application to SARS-CoV-2 spike protein. Front Mol Biosci. (2021) 8:658687. doi: 10.3389/fmolb.2021.658687 34026833 PMC8131536

[34] Weimann A Dinan AM Ruis C Bernut A Pont S Brown K . Evolution and host-specific adaptation of Pseudomonas aeruginosa. Science. (2024) 385:eadi0908. doi: 10.1126/science.adi0908 38963857 PMC7618370

