# Supplementary material for: Structured peptides as vaccine antigens against infections
Source: Front Immunol. 2026 Aug 7;17:1908831. doi: 10.3389/fimmu.2026.1908831 (PMC13493254; doi:10.3389/fimmu.2026.1908831)
Supplement: Supplementary file 1 [file DataSheet1.pdf]

Supporting information

Figure legends.

Figure S1. Secondary structure analysis was performed for the three simulation replicas of the trpzip2PAK peptide. The legend indicates the secondary structure corresponding to each colour.

Table S1: Summary of restraints and structure quality factors of the structure family trpzip2PAK (20 conformers)

Total number of meaningful NOE upper distance constraints[a]	395
Short-range, |i-j|<=1, NOEs	271
Medium-range, 1<|i-j|<5, NOEs	72
Long-range, |i-j|>=5, NOEs	52
Total meaningful dihedral angle restraints	
Phi	10
Psi	10
Chi1	9
Restraints violated in 6 or more structures:	
Distance restraints	0
Van der Waals restraints	2
Angle restraints	0
Average RMSD to the mean (Å)	
Average backbone RMSD to mean	0.68 +/- 0.24
Average backbone RMSD to mean (residues 2-5, 8-12)	0.04 +/- 0.02
Average CYANA Target Function ($Å^2$)	0.55 ± 0.01
Ramachandran plot summary from Procheck[b]	
Most favoured regions	82.8 (66.4) %
Additional allowed regions	17.2 (31.9) %
Generously allowed regions	0.0 (1.7) %
Disallowed regions	0.0 (0.0) %
Ramachandran Plot statistics from Richardson's lab	
Most favoured regions	100.0 (80.0) %
Allowed regions	0.0 (15.8) %
Disallowed regions	0.0 (4.2) %
Structure quality Factors-overall statistics[b]	
Procheck G-factor Z score (phi-psi)	-3.74
Procheck (all)	-6.68
MolProbity Clashscore Z score	-0.24

Note: Structure calculations were performed with the program CYANA (28) 3.98.15. A total of 100 random conformers were subjected to 10,000 steps of simulated annealing. The mean value and the standard deviation are given.

[a]Number of meaningful constraints for each class.

[b]Selected regions ranges:2-5, 8-12. Data in parentheses are obtained considering all residues. Calculated using PSVS (34)
